# Sexual health risk indicators and their associations with caries status and gingival health of adolescents resident in sub-urban South-West Nigeria

**DOI:** 10.12688/aasopenres.13301.1

**Published:** 2022-02-11

**Authors:** Morenike Oluwatoyin Folayan, Maha El Tantawi, Randa Yassin, Olaniyi Arowolo, Nadia A. Sam-Agudu

**Affiliations:** 1 Obafemi Awolowo University, Ile-ife, Nigeria, Ile-Ife, Nigeria, 22005, Nigeria; 2 Department of Pediatric Dentistry and Dental Public Health,, Faculty of Dentistry, Alexandria University,, Alexandria, Egypt; 3 Department of Child Dental Health, Obafemi Awolowo University Teaching Hospitals Complex, Ile-Ife, Nigeria; 4 International Research Center of Excellence,, Institute of Human Virology Nigeria, Abuja, Nigeria; 5 Department of Pediatrics, University of Maryland School of Medicine,, Baltimore, USA

**Keywords:** Adolescents, Risk-taking, Sexual health, Oral Health, Nigeria

## Abstract

**Background:** Adolescents are at high risk of poor sexual and oral health. We investigated for sexual risk factors associated with caries experience and gingival health among adolescents in Nigeria.

**Methods:** This cross-sectional study collected data from 10-19-year-old adolescents in Ile-Ife, South-West Nigeria through a household survey conducted between December 2018 and January 2019. Information collected included age; sex; socioeconomic status; sexual practices (vaginal, oral, anal sex); sexual (transactional sex, multiple sex partners, condom use at last sexual intercourse) and oral health (frequency of tooth brushing, use of fluoridated toothpaste, dental service utilization in the last 12 months, consumption of refined carbohydrates in-between meals) risk behaviors; caries experience; and gingival health. Logistic regression was used to determine associations between explanatory variables (sexual and oral health risk behaviors) and outcome variables (caries experience and gingivitis).

**Results:** There were no significant associations between caries experience and history of sexual intercourse (OR:1.00); condom use at last sex act (OR:0.68); and having one (OR:2.27) or more sexual partners. Also, there was no significant association between moderate/severe gingivitis and a history of anal (OR:2.96), oral (OR:2.69), or vaginal (OR:1.40) sex; and a report of having one (OR:1.71) or more (OR:2.57) sex partners.

**Conclusions:** Some sexual health risk indicators insignificantly increase the risk for caries and moderate/severe gingivitis. Screening for sexual risk behaviors during dental care may be a suitable wellness programs approach for adolescents.

## Abbreviations

AOR          Adjusted Odds Ratio

CI              Confidence Interval

## Introduction

Adolescence increases the risk for periodontal disease and gingival inflammation, due to multiple hormonal changes ^1^. It is also a period where adolescents experiment with sex, and a high proportion of adolescents experience oral sex as their first exposure to sexual activity ^2– 5^. Additionally, higher proportions of adolescents have had oral than vaginal sex, possibly due to myths about the safety of oral sex ^6^. Oral sex is associated with several sexually transmitted infections in both heterosexual ^7– 9^ and same-sex relationships ^6, 10, 11^. Although the risk of orally transmitted infections is less than that of vaginal and anal sex, the risk is not zero. Infections are transmitted in the oral cavity through sores, abrasions, and periodontal disease, and caries creates sharp tooth margins, which can cause cuts in the oral mucosa ^12, 13^. Oral sex may increase the risk of gingival disease either through introduction of microbes or mechanical trauma to the oral cavity ^12^. Vaginal and anal sex are also typically associated with concomitant oral sex and may therefore be indirectly associated with a higher risk of gingival disease ^6^.

Sexually-active adolescents are more likely to use contraception ^14^. However, the use of contraception is associated with oral health complication. Hormonal shifts during menstruation and with oral pills increase the risk of recurrent ulcers and xerostomia, which in turn increases the risk of caries injectable progesterone may also contribute to periodontal disease and gingivitis among women ^15– 17^. Condoms are the most common form of contraception used by adolescents in Nigeria ^18^. They are the most readily accessible contraception in communities where support for, and provision of contraception services for adolescents is poor ^19^. Oral contact with latex condoms may cause allergic reactions in the mouth ^20^, although this risk is likely to be very low as condom use during oral sex is low ^21^ and much lower than it is for anal and vaginal sex ^22^.

Folayan *et al.* ^23^ described a conceptual framework inter-linking the oral, mental, sexual and reproductive health of adolescents and proposed the integration of these services in a one-stop-shop model for delivery in Nigeria. There are very few empirical studies evaluating relationships between behavioral risk factors for poor sexual health and poor oral health among adolescents. In Nigeria, risky sexual behaviors are having multiple sex partners, no/inconsistent use of contraception and engaging in transactional sex ^24– 26^. The risk factors for poor oral health-including caries and periodontal disease are poor tooth-brushing habits, high consumption of refined carbohydrates in-between-meals, and inadequate use of fluoridated toothpaste ^27^. It is possible that poor oral and sexual health may reflect the risk-taking propensity of adolescents as they developmentally transition into adulthood ^28^.

This research builds on the behavioral decision-making framework, which acknowledges that adolescents do not consider themselves vulnerable ^29^. Rather, social and affective factors can influence their behavior through decision-making processes and influence how they discount future outcomes. We also acknowledge that cognitive control (inhibition) increases with age across childhood and adolescence, and that this increase is associated with maturation of the prefrontal cortex, which also influences rational decision-making ^30^. Social and affective factors affecting risk- taking also vary. For example, older adolescents ^31^, females ^32– 34^ are less likely to take risks. There may be associations between risk taking behavior and socioeconomic status but these associations are however, not as robust as that found in adults ^32, 35^.

Risk-taking behavior may impact all aspects of life, with high risk-takers likely to fare poorer in overall health compared to low risk-takers. We therefore sought to evaluate for associations between sexual and oral health risk-taking behaviors and outcomes of poor oral health, with a focus on identifying sexual risk factors associated with caries and gingival health. We hypothesized that sexual health risk behaviors will be associated with poor oral health (caries experience and moderate to severe gingivitis).

## Methods

### Study population and study design

Data was collected through a household survey conducted between December 2018 and January 2019 in Ife Central Local Government Area of Ile-Ife, a semi-urban community in Osun State, South-West Nigeria. Adolescents aged 10–19 years and from whom parental consent and individual assent, or individual informed consent were obtained where appropriate, were eligible to participate. Adolescents who had severe mental health conditions or were critically ill and could not give independent responses to the study survey were excluded. Recruitment of participants continued until the sample size for the study was reached.

### Sample size and sampling technique

The minimum sample size was calculated with the formula proposed by Araoye ^36^. With a caries prevalence of 13.9% among adolescents in the study setting ^37^, a margin of error of 5%, and a confidence level of 95%, the minimum sample size was 1,323 adolescents. Participants were recruited with a multi-stage sampling technique. First, 70 of the 700 enumeration areas in Ife Central Local Government Area were sampled with the simple random technique. Next, every other household in the selected enumeration areas was identified as eligible. Finally, in each household, one adolescent who met inclusion criteria was recruited for the study. Whenever a household declined to participate, the next eligible household was substituted.

### Data variables

***Demographic, sexual practices and sex behavior profile:*** Information on socio-demographics (age, sex, socioeconomic status), sexual practices (vaginal, oral and anal sexual intercourse), and risky sexual behavior (transactional sex, multiple sex partners use of condom at last sexual intercourse) were collected. Assessment of sexual health factors was done using a questionnaire validated for use in the 2007 National HIV and AIDS Reproductive Health Survey of Nigeria ^38^. For the purpose of this study, specific questionnaire items used to capture information were those relating to vaginal, anal and oral sex practices, multiple sexual partnering, unprotected sex and transactional sex.

***Socioeconomic status.*** Data on socioeconomic status were determined by an adapted version of the index developed by Olusanya *et al.* ^39^, which has been used in a previous survey in our selected study setting ^40^. This is a multiple-item index combining maternal level of education with paternal educational level and occupation. Each adolescent was allocated into social classes I–V (Class I: upper class; Class II: upper middle class; Class III: middle class; Class IV, lower middle class; Class V, lower class). These were combined for the analysis into: Class I (upper and upper middle classes), Class II (middle class) and Class III (lower middle and lower classes). For adolescents who had lost a parent, socioeconomic status was determined using the status of the living parent; primary caregiver data was used for those who had lost both parents.

***Tooth brushing.*** Respondents were asked to indicate the frequency of tooth brushing using the following alternatives – irregularly or never, once a week, a few (2–3) times a week; once a day, and more than once a day. Respondents were classified into those who brushed more than once a day (at least twice daily) and “Others”, including those who brushed ‘irregularly or never, once a week, a few (2–3) times a week; once a day’ were classified as not having undertaken preventive dental care ^41^.

***Use of fluoridated toothpaste.*** Respondents indicated the frequency of use of fluoridated toothpaste when tooth brushing, using the following alternatives – *Always*, *quite often*, *seldom*, *not at all*. Respondents were divided into those who always used fluoridated toothpaste (Always) and those who did not always use fluoridated toothpaste (choosing the options ‘ *quite often*, *seldom*, *not at all*’) ^41^.

***Consumption of refined carbohydrates in-between-meals.*** Respondents indicated the frequency of consuming sugar-containing snacks or drinks between main meals using the following alternatives – About three times a day or more, about twice a day, about once a day, occasionally; not every day, rarely, or never between meals. Respondents who chose the options ‘About three times a day or more, about twice a day, about once a day’, were classified as consuming refined carbohydrates in between meals daily ^41^.

***Dental service utilization.*** Respondents indicated time of the last dental check-up using the following alternatives - within the last six months, more than six months to one year ago, more than one to two years ago, more than two to five years ago, more than five years, never, do not remember. Participants were classified into those attending a dental check-up within the last year and those who did not (choosing the options ‘more than one to two years ago, more than two to five years ago, more than five years, never or do not remember’) ^41^.

***Caries.*** Caries experience was assessed as the sum of the decayed, missing, and filled teeth (DMFT) index, using the World Health Organization criteria ^42^. Caries was assessed using a disposable mirror and explorer under natural daylight, with participants seated, without magnification, drying or radiographs. Caries experience was further divided into ‘present’ (DMF> 0) or ‘absent’ (DMF= 0).

***Plaque index.*** The plaque index was used to determine oral hygiene status. Plaque index score was based on six numerical determinations representing the amount of debris found on the surfaces of index permanent teeth 12, 16, 24, 32, 36, and 44. The mesial, distal, buccal, and lingual gingival areas of the index teeth are scored from 0 (no plaques) to 3 (abundance of soft matter within the gingival pocket and/or on the tooth and gingival margin). The mean score for each tooth was obtained, and the mean score for the individual was determined by adding the indices for each tooth and dividing by the number of teeth examined.

***Gingival health.*** The presence and severity of gingivitis was evaluated with the gingival index, as described by Löe and Silness. Changes in the gingiva in six index teeth (7, 3, 12, 19, 23 and 28) in the permanent dentition were assessed. Four areas of each index tooth were scored, and the scores were summed and divided by four to give the gingival index for each tooth. The gingival index for each participant was obtained by adding the values of all index teeth and dividing by six. Gingivitis was classified as healthy, mild, moderate, or severe, with values of <0, 0.1—1, 1.1—2, and 2.1–3, respectively. Gingivitis was dichotomized into healthy and mild gingivitis versus moderate-to-severe gingivitis ^43^.

### Data analysis

Descriptive analysis was performed to determine the proportion of male and female adolescents with sociodemographic variables, oral and sexual health risk indicators. Males and females were compared using chi square or Fisher’s exact tests as indicated. Bivariate analysis, followed by univariate logistic regression analysis (unadjusted) were used to determine the association between the explanatory variables (sexual and oral health behavior and practices) and the outcome variables (caries experience and gingival health). This was followed by the construction of adjusted models which included all variables, with and without history of sexual intercourse and adjusting for sociodemographic variables. The estimated coefficients, expressed as adjusted odds ratios (AOR) and their 95% confidence intervals, were calculated. Statistical analysis was conducted using IBM SPSS Statistics for Windows, version 22 (IBM Corp., Armonk, N.Y., USA). Statistical significance was inferred at p <0.05.

### Ethical considerations

Ethical approval for the study was obtained from the Ethics and Research Committee of the Institute of Public Health, Obafemi Awolowo University, Ile-Ife, Nigeria [IPHOAU/12/669]. Approval for conduct of the study was obtained from the Local Government Authority prior to commencement. The study was conducted in full compliance with the protocol. Informed consent was obtained from the parent/adult caregiver of each study participant aged 10 – 11 years old prior to enrollment. Parental consent and participant assent was obtained for those 12–13 years old. Consent was obtained from study participants aged 14 to 19 years in line with guidance from the Federal Ministry of Health ^44^. Efforts were made to minimize risks and loss of confidentiality for participants by ensuring that data collection was conducted privately and anonymized, via an electronic data platform. Study participants’ discomfort with the personal nature of questions was mitigated by ensuring that field workers were trained on how to ask sensitive questions and to clarify non-verbal cues observed during the interviews. No compensation was paid to adolescents for study participation.

## Results

Complete responses for the 1,244 participants ^45^ of which 701 (56.4%) were male and 437 (35.1%) had high socioeconomic status ( Table 1). The mean age (standard deviation) of study participants was 14.6 (2.7) years, and the mean plaque index score was 0.82. Only 110 (8.8%) participants brushed their teeth at least twice daily, 1135 (91.2%) reported using fluoridated toothpaste, 771 (62.0%) consumed refined carbohydrates in-between meals daily, and 14 (1.1%) had visited the dentist in the last 12 months. Additionally, 91 (7.3%) reported a history of sexual intercourse. The male cohort was significantly older than the female cohort in this study (14.8 vs 14.4 years; *p*= 0.01). A lower proportion of males than females reported brushing their teeth at least twice daily (7.3% vs 10.9%; *p*= 0.03).

**Table 1.** Demographic profile, oral health indicators and history of sexual intercourse among adolescents, by sex (N= 1,244).

Factors	Male 701 (56.4%)	Female 543 (43.6%)	p value	Total 1244 (100%)
Age				
Mean (SD)	14.8 (2.7)	14.4 (2.6)	0.01 *	14.6 (2.7)
Socioeconomic status				
High	256 (36.5)	181 (33.3)	0.40	437 (35.1)
Middle	238 (34.0)	185 (34.1)		423 (34.0)
Low	207 (29.5)	177 (32.6)		384 (30.9)
Toothbrushing at least twice daily				
Yes	51 (7.3)	59 (10.9)	0.03 *	110 (8.8)
No	650 (92.7)	484 (89.1)		1134 (91.2)
Use of fluoridated toothpaste				
Yes	634 (90.4)	501 (92.3)	0.26	1135 (91.2)
No	67 (9.6)	42 (7.7)		109 (8.8)
Plaque index				
Mean (SD)	0.83 (0.56)	0.81 (0.57)	0.64	0.82 (0.56)
Daily consumption of refined carbohydrates in between meals				
Yes	427 (60.9)	344 (63.4)	0.38	771 (62.0)
No	274 (39.1)	199 (36.6)		473 (38.0)
Dental service utilization in 12 months				
Yes	5 (0.7)	9 (1.7)	0.17	14 (1.1)
No	696 (99.3)	534 (98.3)		1230 (98.9)
Ever had sex				
Yes	49 (7.0)	42 (7.7)	0.62	91 (7.3)
No	652 (93.0)	501 (92.3)		1153 (92.7)

* *Statistically significant at p< 0.05*

Table 2 shows that 7 (7.7%) of the 91 adolescents with a history of sexual intercourse reported a history of transactional sex, 12 (13.2%) had anal sex, 70 (76.9%) had vaginal sex and 18 (19.8%) had oral sex. Also, most (52.7%) of those with a history of sexual intercourse reported having one partner, and 33 (58.9%) reported using condoms at the last sex act. There were no statistically significant sex (male vs female) differences in reported sexual risk behaviors of study participants.

**Table 2.** Differences in sexual activity characteristics between male and female adolescents (N= 91).

Factors	Male 49 (53.8%) n (%)	Female 42 (46.2%) n (%)	p value	Total 91 (100%) n (%)
History of transactional sex				
Yes	3 (6.1)	4 (9.5)	0.70	7 (7.7)
No	46 (93.9)	38 (90.5)		84 (92.3)
Anal sex				
Yes	7 (14.3)	5 (11.9)	0.74	12 (13.2)
No	42 (85.7)	37 (88.1)		79 (86.8)
Vaginal sex				
Yes	39 (79.6)	31 (73.8)	0.51	70 (76.9)
No	10 (20.4)	11 (26.2)		21 (23.1)
Oral sex				
Yes	10 (20.4)	8 (19.0)	0.87	18 (19.8)
No	39 (79.6)	34 (81.0)		73 (80.2)
Number of current sex partners				
0	16 (32.7)	10 (23.8)	0.26	26 (28.6)
1	22 (44.9)	26 (61.9)		48 (52.7)
More than 1	11 (22.4)	6 (14.3)		17 (18.7)
Use of condom at last sex ^¶^				
Yes	18 (56.3)	15 (62.5)	0.64	33 (58.9)
No	14 (43.8)	9 (37.5)		23 (41.1)

*^¶^: N= 56*

Table 3 shows that adolescents with caries experience were significantly older than those without caries experience (15.6 years vs 14.6 years; *p*= 0.02). In the fully adjusted model, caries experience was significantly associated with older age (AOR: 1.18; 95% CI: 1.04, 1.34) and inversely associated with socioeconomic status: there were lower odds of caries experience among adolescents of high socioeconomic status, compared to those from low socioeconomic status (AOR: 0.44; 95% CI: 0.20, 0.96). There was no association between the presence of caries experience and history of sexual intercourse (AOR: 1.00; 95% CI: 0.36, 2.77)

**Table 3.** Sociodemographic characteristics, oral health indicators and sexual history associations with caries experience (N= 1,244).

Factors	Caries experience		p value	OR (95% CI)	AOR1 (95% CI)	AOR2 (95% CI)
	Present 46 (3.7%)	Absent 1198 (96.3%)				
Age						
Mean (SD)	15.6 (2.7)	14.6 (2.7)	0.02 *	1.15 (1.03, 1.29) *	1.18 (1.05, 1.33) *	1.18 (1.04, 1.34) *
Sex						
Male	28 (60.9)	673 (56.2)	0.53	1.21 (0.66, 2.22)	1.19 (0.65, 2.19)	1.19 (0.65, 2.19)
Female	18 (39.1)	525 (43.8)		1.00	1.00	1.00
Socioeconomic status						
High	12 (26.1)	425 (35.5)	0.34	0.57 (0.27, 1.21)	0.45 (0.21, 0.98) *	0.44 (0.20, 0.96) *
Middle	16 (34.8)	407 (34.0)		0.80 (0.40, 1.59)	0.73 (0.36, 1.46)	0.73 (0.36, 1.47)
Low	18 (39.1)	366 (30.6)		1.00	1.00	1.00
Toothbrushing at least twice daily						
Yes	4 (8.7)	106 (8.8)	1.00	0.98 (0.35, 2.79)	1.14 (0.40, 3.30)	1.14 (0.40, 3.30)
No	42 (91.3)	1092 (91.2)		1.00	1.00	1.00
Use of fluoridated toothpaste						
Yes	42 (91.3)	1093 (91.2)	1.00	1.01 (0.36, 2.87)	1.15 (0.40, 3.32)	1.15 (0.40, 3.33)
No	4 (8.7)	105 (8.8)		1.00	1.00	1.00
Plaque index						
Mean (SD)	0.80 (0.54)	0.82 (0.56)	0.82	0.94 (0.55, 1.59)	0.92 (0.54, 1.57)	0.92 (0.54, 1.57)
Daily consumption of refined carbohydrates in between meals						
Yes	29 (63.0)	742 (61.9)	0.88	1.05 (0.57, 1.93)	0.97 (0.52, 1.81)	0.97 (0.52, 1.81)
No	17 (37.0)	456 (38.1)		1.00	1.00	1.00
Dental service utilization in 12 months						
Yes	0 (0)	14 (1.2)	1.00	0.00 (0.00,-)	0.00 (0.00,-)	0.00 (0.00,-)
No	46 (100)	1184 (98.8)		1.00	1.00	1.00
Ever had sex						
Yes	5 (10.9)	86 (7.2)	0.38	1.58 (0.61, 4.09)	-	1.00 (0.36, 2.77)
No	41 (89.1)	1112 (92.8)		1.00	-	1.00

* *Statistically significant at p< 0.05*

Table 4 shows that condom use in the last sex act was associated with lower odds of caries experience when compared with no condom use (OR= 0.68), although the association was not statistically significant (95% CI: 0.09, 5.19) ( Table 4). Furthermore, when compared to adolescents reporting having no sex partners in the last 12 months, those who had one partner had higher odds of caries experience (OR= 2.27) although the association was not statistically significant (95% CI: 0.24, 21.47).

**Table 4.** Associations between sexual risk behaviors and caries experience (N=91).

Factors	Caries experience		p value	OR (95% CI)
	Present 5 (5.5%)	Absent 86 (94.5%)		
Sexual practices: anal				
Yes	0 (0)	12 (14.0)	1.00	0.00 (0.00, -)
No	5 (100)	74 (86.0)		1.00
Sexual practices: oral				
Yes	0 (0)	18 (20.9)	0.58	0.00 (0.00, -)
No	5 (100)	68 (79.1)		1.00
Sexual practices: vaginal				
Yes	5 (100)	65 (75.6)	0.59	4.9 x 10 ^9^ (0.00, -)
No	0 (0)	21 (24.4)		1.00
Transactional sex				
Yes	0 (0)	7 (8.1)	1.00	0.00 (0.00, -)
No	5 (100)	79 (91.9)		1.00
Used condom at last sex act				
Yes	2 (50.0)	31 (59.6)	1.00	0.68 (0.09, 5.19)
No	2 (50.0)	21 (40.4)		1.00
Number of sex partners				
None	1 (20.0)	25 (29.1)	0.39	1.00
One	4 (80.0)	44 (51.2)		2.27 (0.24, 21.47)
More than one	0 (0)	17 (19.8)		0.00 (0.00, -)

Table 5 shows that in the fully adjusted model, using fluoridated toothpaste (AOR: 3.32; 95% CI: 1.08, 10.26) and higher plaque index score (AOR: 13.86 95% CI: 8.89, 21.62) were associated with significantly higher odds of moderate to severe gingivitis among adolescents with a sexual history; a finding similar to those without a sexual history (AOR: 13.87 95% CI: 8.90, 21.63). Daily consumption of refined carbohydrates in-between-meals was associated with significantly lower odds of moderate to severe gingivitis when compared with non-daily consumption of refined carbohydrates in-between-meals for adolescents with and without a sexual history (AOR: 0.50, 95% CI: 0.31, 0.80) respectively.

**Table 5.** Sociodemographic characteristics, oral health indicators and sexual history associations with moderate to severe gingivitis (N=1,244).

Factor	Moderate/ severe gingivitis		p value	OR (95% CI)	AOR1 (95% CI) (without sexual history)	AOR2 (95% CI) (with sexual history)
	Present 46 (3.7%)	Absent 1198 (96.3%)				
Age						
Mean (SD)	14.72 (2.65)	14.62 (2.66)	0.09	1.03 (0.94, 1.09)	1.02 (0.93, 1.12)	1.02 (0.92, 1.12)
Sex						
Male	65 (59.6)	636 (56.0)	0.47	1.16 (0.78, 1.73)	1.13 (0.71, 1.80)	1.13 (0.71, 1.80)
Female	44 (40.4)	499 (44.0)		1.00	1.00	1.00
Socioeconomic status						
High	36 (33.0)	401 (35.3)	0.76	0.84 (0.52, 1.36)	0.96 (0.53, 1.74)	0.96 (0.53, 1.74)
Middle	36 (33.0)	387 (34.1)		0.87 (0.54, 1.41)	0.98 (0.56, 1.72)	0.98 (0.56, 1.73)
Low	37 (33.9)	347 (30.6)		1.00	1.00	1.00
Toothbrushing at least twice daily						
Yes	11 (10.1)	99 (8.7)	0.63	1.18 (0.61, 2.27)	0.98 (0.45, 2.11)	0.97 (0.45, 2.10)
No	98 (89.9)	1036 (91.3)		1.00	1.00	1.00
Use of fluoridated toothpaste						
Yes	105 (96.3)	1030 (90.7)	0.05	2.68 (0.97, 7.41)	3.34 (1.08, 10.31) *	3.32 (1.08, 10.26) *
No	4 (3.7)	105 (9.3)		1.00	1.00	1.00
Plaque index						
Mean (SD)	1.56 (0.53)	0.75 (0.51)	0.81	13.54 (8.78, 20.87) *	13.87 (8.90, 21.63) *	13.86 (8.89, 21.62) *
Daily consumption of refined carbohydrates in between meals						
Yes	56 (51.4)	715 (63.0)	0.02 *	0.62 (0.42, 0.92) *	0.50 (0.31, 0.80) *	0.50 (0.31, 0.80) *
No	53 (48.6)	420 (37.0)		1.00	1.00	1.00
Dental service utilization in 12 months						
Yes	3 (2.8)	11 (1.0)	0.12	2.89 (0.79, 10.53)	1.33 (0.28, 6.25)	1.32 (0.28, 6.22)
No	106 (97.2)	1124 (99.0)		1.00	1.00	1.00
Ever had sex						
Yes	11 (10.1)	80 (7.0)	0.24	1.48 (0.76, 2.87)	-	1.14 (0.47, 2.75)
No	98 (89.9)	1055 (93.0)		1.00	-	1.00

*: Statistically significant at p< 0.05

Table 6 indicates that although there were no significant associations between sexual risk behaviors and moderate to severe gingivitis, a history of anal (OR: 2.96; 95% CI: 0.66, 13.22), oral (OR: 2.69; 95% CI: 0.69, 10.47), and vaginal sex (OR:1.40; 95% CI: 0.28, 7.06); and a report of having one (OR:1.71; 95% CI: 0.32, 9.17) or more (OR:2.57; 95% CI: 0.38, 17.31) sex partners was associated with higher odds of moderate to severe gingivitis. 

**Table 6.** Association between sexual risk behaviors and moderate to severe gingivitis (N=91).

Factors	Moderate to severe gingivitis		*p* value	OR (95% CI)
	Present 49 (53.8%)	Absent 42 (46.2%)		
Sexual practice: anal				
Yes	3 (27.3)	9 (11.3)	0.16	2.96 (0.66, 13.22)
No	8 (72.7)	71 (88.8)		1.00
Sexual practice: oral				
Yes	4 (36.4)	14 (17.5)	0.22	2.69 (0.69, 10.47)
No	7 (63.6)	66 (82.5)		1.00
Sexual practice: vaginal				
Yes	9 (81.8)	61 (76.3)	1.00	1.40 (0.28, 7.06)
No	2 (18.2)	19 (23.8)		1.00
Transactional sex				
Yes	0 (0)	7 (8.8)	0.59	0 (0, -)
No	11 (100)	73 (91.3)		1.00
Used condom at last sex act				
Yes	3 (100)	30 (56.6)	0.26	6.3 x 10 ^8^ (0, -))
No	0 (0)	23 (43.4)		1.00
Number of sex partners				
None	2 (18.2)	24 (30.0)	0.61	1.00
One	6 (54.5)	42 (52.5)		1.71 (0.32, 9.17)
More than one	3 (27.3)	14 (17.5)		2.57 (0.38, 17.31)

## Discussion

In our cohort of adolescents resident in South-West Nigeria, we did not identify any sexual health risk-taking behavior that was a significant risk indicator for caries and moderate/severe gingivitis. The study hypothesis was thus, not supported. However, we found that having one sex partner in the last year was associated with higher odds of having caries and moderate/severe gingivitis compared to not having a partner; condom use in the last sex act was associated with lower odds of caries experience; having more than one sex partner was associated with higher odds of having moderate/severe gingivitis compared to those without a partner; and having anal, vaginal and oral sex was associated with higher odds of having moderate/severe gingivitis.

Our study provides some new insight into the possible associations between sexual health and oral health among adolescents. Although the large sample size allowed for robust analysis, the low caries prevalence in this study cohort may have reduced the probability of finding significant associations between sexual health risk factors and caries. In addition, the low number of sexually active respondents also resulted in the wide confidence intervals. The low proportion of respondents reporting sexual activity – a proportion lower than the 14.9% reported for Osun State (the state with the lowest proportion of adolescents reporting sexual intercourse in Nigeria) in 2013 ^46^ – may result from social desirability bias. Pre-marital sex is highly frowned upon in Nigerian society, and thus, despite best possible efforts to reduce the risk of social desirability bias by carefully wording survey questions and engaging young data collectors, this problem may not have been completely eliminated. Furthermore, this is a cross sectional study, and although we attempted to establish an association between oral health and sexual health risk indicators, we do not claim to have established a causal relationship between the variables.

Despite the study limitations, we provide evidence of a possible complex relationship between oral and sexual health in adolescents. For example, the risk of caries increases with increasing age ^47^, while risk-taking behaviors in adolescence decrease with increasing age ^30^. On the contrary, the risk for behaviors leading to poor oral health is higher among adolescents with low socioeconomic status, just like it is for sexual risk-taking behaviors, especially among young females ^48^. In effect, age, gender and socioeconomic status may be mediators and/or moderators of risk-taking behaviors among adolescents; and it may be possible to explore the mediating and/or moderating roles of these characteristics in the relationship between adolescent oral and sexual health in future studies.

We found no association between caries experience, and moderate to severe gingivitis and sexual health risk behaviors. The opposite was true for Cunha *et al.* ^49^ who found an increase in oral lesions among Brazilian adolescents and young people who had multiple sex partners and had condomless sex. While we found no association between oral disease and sexual health risk behaviors, the high odds of adolescents with oral diseases having sexual health risk behaviors suggest there is a place and importance for sexual health screening among adolescents who present at oral health clinics. The Home, Education/employment, peer group Activities, Drugs, Sexuality, and Suicide/depression (HEADSS) psychosocial assessment tool ^50^ for comprehensive history-taking for adolescents provides an opportunity to appropriately assess sexual health in dental clinics. Such an integrated approach for adolescent care will help promote early diagnosis, prompt access to preventive and supportive services, and reduced sexual health risks for those who access the dental clinic. Dental health personnel would need to imbibe adolescent-friendly behaviors to be able to elicit sex and sexuality-related histories, as discussions about sexual health are sensitive issues for many young people in Nigeria, due to cultural norms, religious barriers, embarrassment or peer pressure ^51^.

Although the study finding showed that moderate to severe gingivitis among adolescents was associated with higher plaque index score (not an unexpected finding), there was also higher odds for moderate to severe gingivitis when using fluoridated toothpaste; and lower odds for moderate to severe gingivitis with daily consumption of refined carbohydrates in-between-meals (unexpected findings). Prior studies indicated that toothpaste containing triclosan/copolymer or stannous fluoride reduce the risk for gingivitis ^52, 53^. High sugar consumption is also associated with poor periodontal health in adolescents ^54^ by triggering a hyperinflammatory state ^55^. The findings of this study, therefore, needs to be explored further.

## Conclusion

Within the limitations of the low prevalence of oral disease and risk indicators for sexual health observed in our Nigerian cohort, findings suggest that some sexual health risk indicators may be associated with caries experience and moderate to severe gingivitis among adolescents. However, history of sexual intercourse seems to only be weakly associated with oral disease. Future research is needed to further elucidate mediating and/or moderating roles of age, gender and socioeconomic status in the relationship between adolescent oral and sexual health. Lastly, integrating the screening of sexual health problems within regular dental care service delivery for adolescents may help in the early identification of oral and sexual health issues among adolescents. 

## Data availability

### Underlying data

Figshare: Supplemental file 1: Sexual health risk indicators and their associations with caries status and gingival health of adolescents resident in sub-urban South-West Nigeria, https://doi.org/10.6084/m9.figshare.16607870.

This project contains the sexual health and oral health dataset.xlsx

Data are available under the terms of the Creative Commons Attribution 4.0 International license (CC-BY 4.0).
