# Supplementary material for: Sexual health risk indicators and their associations with caries status and gingival health of adolescents resident in sub-urban South-West Nigeria
Source: AAS Open Res. 2022 Feb 11;5:7. [Version 1] doi: 10.12688/aasopenres.13301.1 (PMC11287114; doi:10.12688/aasopenres.13301.1)
Supplement: Peer review history

# Reviewer report: Reviewer response for version 1

**DOI:** 10.21956/aasopenres.14427.r30033

**Published:** 2023-08-24

**Authors:** Cennikon Pakpahan

**Affiliations:** 1 Universitas Airlangga, Surabaya, East Java, Indonesia

This an interesting paper to explore sexual health behavior correlates with oral health with many respondents. I appreciate the effort of the authors to conduct the study.

 The results illustrate no correlation between sexual behavior and oral hygiene, especially the prevalence of carries. I think these results are caused by multiple factors that influenced the caries. Even after the authors performed an adjustment, still the results show no significant correlation.

 Still, there is space for improvement in the paper:

- The introduction needs some additions such as literature that stated the phenomenon of sexual behavior among the adolescents, the contribution of popular culture etc. 
- Other than that I do not agree with the table explanation which states the AOR value even though it is not statistically significant. So I prefer the explanation or that the legend be revised.
- The discussion also needs some improvement, such as elucidating why there is no correlation between the parameters. 

Is the work clearly and accurately presented and does it cite the current literature?

Partly

If applicable, is the statistical analysis and its interpretation appropriate?

Partly

Are all the source data underlying the results available to ensure full reproducibility?

Yes

Is the study design appropriate and is the work technically sound?

Partly

Are the conclusions drawn adequately supported by the results?

Yes

Are sufficient details of methods and analysis provided to allow replication by others?

Yes

Reviewer Expertise:

Sexual and Reproductive Health

I confirm that I have read this submission and believe that I have an appropriate level of expertise to confirm that it is of an acceptable scientific standard, however I have significant reservations, as outlined above.

# Response

**Published:** 2024-02-14

**Authors:** Morenike Oluwatoyin Folayan

**Affiliations:** Obafemi Awolowo University, Nigeria

This an interesting paper to explore sexual health behavior correlates with oral health with many respondents. I appreciate the effort of the authors to conduct the study. Response: Thanks for the constructive feedback provided. This has helped to strengthen the manuscript. We have addressed the concerns raised by the author. Please find below the point-by-point response to the issues raised. 

 The results illustrate no correlation between sexual behavior and oral hygiene, especially the prevalence of carries. I think these results are caused by multiple factors that influenced the caries. Even after the authors performed an adjustment, still the results show no significant correlation. Still, there is space for improvement in the paper:

- The introduction needs some additions such as literature that stated the phenomenon of sexual behavior among the adolescents, the contribution of popular culture etc.

Response: Thanks for this. We have included two paragraphs in the introduction to discuss these issues and link them with the focus of the study. Please see paragraphs 2 and 3 (references 14-23) in the introduction section of the manuscript.

- Other than that I do not agree with the table explanation which states the AOR value even though it is not statistically significant. So I prefer the explanation or that the legend be revised.

Response: We have revised the report of the results to reflect the non-significance of the associations observed. We have reduced the number of tables from 6 to 5 as the prior Table 2 was not linked with the study objectives. 

- The discussion also needs some improvement, such as elucidating why there is no correlation between the parameters.

Response: we have improved the discussion by outlining the padadox in the research findings, and the oral health policy and program implications of the study findings. Please see the last paragraph in the discussion section of the manuscript.
